# Capillary coating as an important factor in optimization of the off-line and on-line MEKC assays of the highly hydrophobic enzyme chlorophyllase

**DOI:** 10.1007/s00216-016-0097-5

**Published:** 2016-11-25

**Authors:** Paweł Mateusz Nowak, Michał Woźniakiewicz, Maciej Michalik, Leszek Fiedor, Paweł Kościelniak

**Affiliations:** 1 Faculty of Chemistry, Jagiellonian University in Kraków, Ingardena 3, 30-060 Kraków, Poland; 2 Faculty of Biochemistry, Biophysics and Biotechnology, Jagiellonian University in Kraków, Gronostajowa 7, 30-387 Kraków, Poland

**Keywords:** Capillary electrophoresis, Capillary coating, Chlorophyll, Chlorophyllase, Electrophoretically mediated microanalysis, Micellar electrokinetic chromatography

## Abstract

The choice between bare and coated capillaries is a key decision in the development and use of any methods based on capillary electrophoresis. In this work several permanently and dynamically coated capillaries were successfully implemented in a previously developed micellar electrokinetic chromatography (MEKC) assay of the plant membrane enzyme chlorophyllase. The results obtained demonstrate the rationale behind the use of capillary coating, which is crucial for successful optimization of both the off-line mode and the on-line/electrophoretically mediated microanalysis assay mode. The application of an amine permanently coated capillary (eCAP) is a simple way to significantly increase the repeatability of migration times and peak areas, and to ensure a strong electroosmotic flow that considerably decreases the overall analysis time. A dynamic coating (CEofix) allows one to apply an on-line incubation to control the reaction progress inside the capillary, and to increase the signal-to-noise ratio and peak efficiency. The dynamic coating is possible with use of both the normally applied uncoated silica capillary and the precoated amine capillary, which ensures more repeatable migration times. The strong points of the uncoated silica capillary are its attractive price and wide range of pH that can be applied. The characteristics presented may simplify the choice of capillary modification, especially in the case of hydrophobic analytes, MEKC-based separations, and other enzymatic assays.

**Electronic supplementary material:**

The online version of this article (doi:10.1007/s00216-016-0097-5) contains supplementary material, which is available to authorized users.

## Introduction

Capillary electrophoresis (CE) has become a routine instrumental technique applied in biochemical analysis. Most of the CE-based methods rely on the use of low-cost bare fused-silica capillaries, but there are many reports on the application of coated capillaries, which are recommended for many reasons [1–5]. Capillary coating is a potential method for controlling or eliminating electroosmotic flow (EOF), for preventing undesirable adsorption of analytes onto the capillary inner surface, and for increasing the reproducibility of the migration times, resolution, and peak efficiency. The broad commercial availability of permanently coated capillaries and reagents for reversible dynamic coating makes the choice not straightforward. Intriguingly, only several systematic comparisons of various capillary modifications can be found in the literature [6–13].

There are many reports on CE-based assays of water-soluble enzymes [14–17], which are easier to use than hydrophobic membrane enzymes because of the need to maintain a hydrophobic environment for both the reaction mixture and the separation medium. In the latter case the use of capillary coating is especially advisable to minimize potential undesirable effects; for example, the formation of protein aggregates that may adsorb onto the capillary inner surface, leading to the deterioration of the repeatability of both migration times and peak areas, and to the loss of catalytic activity. Therefore, the development of CE enzymatic assays of membrane proteins in combination with a rational choice of capillary modification is an important direction of research.

**Fig. 1:**
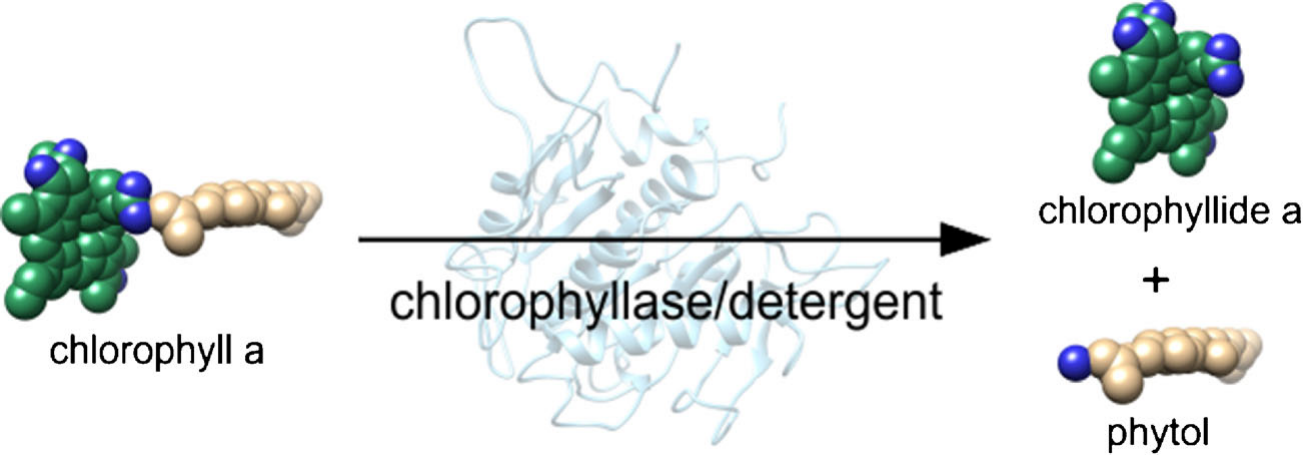
The reaction of chlorophyll hydrolysis catalyzed by chlorophyllase

Chlorophyllase (Chlase) is an ubiquitous plant membrane enzyme catalyzing a hydrolytic cleavage of chlorophyll to chlorophyllide and phytol, which is the first step in chlorophyll biodegradation [18]; see Fig. 1. Its catalytic activity is of global importance to the biosphere [18, 19], but the mechanisms of Chlase regulation in plants are not well understood [19]. Studies on this enzyme are of interest not only for its significant physiological role and the regulatory mechanisms involved in vivo [19] but also for its applications in the olive oil industry [20], and as a convenient synthetic tool in the modification of chlorophyll s as photosensitizers for photodynamic therapy [21, 22]. However, because of its very high hydrophobicity, any experimental work with Chlase is still a challenge. Recently, we developed a CE-based assay of recombinant Chlase from *Arabidopsis thaliana* based on a bare silica capillary as the first such assay of a plant membrane enzyme [23].

A CE-based enzymatic assay may be realized in an off-line mode, where the reaction occurs in a separate vessel, or in an on-line mode, where the mixing of reactants, the reaction, and the separation occur directly in the capillary [24, 25]. The latter approach facilitates automation and a spectacular reduction of reaction volumes, down even to a nanoliter scale. Electrophoretically mediated microanalysis (EMMA) is a variant of the on-line method, and is based on the injection of several plugs containing reagents with different electrophoretic mobilities that are mixed by application of a voltage. After reaction inside the capillary, the substrates and the products are separated as in the off-line mode. Reaction progress can be controlled by the introduction of a zero potential amplification step directly after mixing, without the application of a voltage. In this case, maintenance of resolution between the particular peaks plays a crucial role, and is often problematic because of the strong diffusional effects and the consequent peak broadening.

In this work we present a comparison of several commercially available capillary coatings applied in the analysis of the enzymatic activity of Chlase, and the evaluation of their advantages over the uncoated silica capillary. Both the off-line reaction mode and the on-line reaction mode were tested. Particular attention was paid to analysis of the following criteria: repeatability of migration times and peak areas, selectivity, peak efficiency, signal-to-noise ratio, total analysis time, and feasibility of EMMA with the programmed zero potential amplification step in-capillary [24, 25]. Our results indicate some particular characteristics of the permanently and dynamically coated capillaries that are potentially important in the context of other methods using the CE technique.

## Materials and methods

### Instrumentation

The experiments were performed with a P/ACE MDQ CE system (Beckman Coulter, Brea, CA, USA) equipped with a diode-array detector. The following commercially available capillaries were used: an unmodified bare fused-silica capillary; a neutral eCAP^TM^ polyacrylamide-coated capillary providing neutralization of EOF; an amine eCAP^TM^ polyamine-coated capillary providing the reversal of EOF (Beckman Coulter); and Celerity^TM^ capillary columns coated with chromatographic stationary phases: C_18_, diol, and cholesterol (MicroSolve Technology, Eatontown, NJ, USA). All capillaries were of 60-cm total length, 50-cm effective length, and 50-μm internal diameter. In addition, the silica and amine capillaries were subjected to dynamic coating performed with a commercially available CEofix^TM^ kit (Beckman Coulter). Coating by a double ionic-polymer layer (cationic–anionic) was performed with the silica capillary, and by a single anionic-polymer layer with the amine capillary, precoated permanently by a polyamine layer. Recently, the efficiency of such a specific double coating was examined by us during a search for the optimal capillary type for determination of acid dissociations constant by CE [26].

UV–vis absorption spectra were collected between 200 and 600 nm; 430 nm was the analytical wavelength for the monitoring of the reaction substrate and product. The sample trays and capillaries were conditioned at 30 °C. All aqueous solutions were prepared with deionized water (Milli-Q, Merck Millipore, Billerica, MA, USA), filtered through a 0.45-μm regenerated cellulose membrane, and then degassed by sonication and centrifugation.

The rinsing of capillaries between the runs was done by application of a pressure of 137.9 kPa (20 psi). The procedures either were developed in our laboratory (for the uncoated silica capillary) or followed the instructions provided by the suppliers. The uncoated silica capillary, between the runs, was rinsed with 0.1 M NaOH for 4 min and with the running buffer for 4 min. Before the first use of the capillary on a working day, it was rinsed with methanol for 5 min, 0.1 M HCl for 3 min, deionized water for 3 min, 0.1 M NaOH for 10 min, and the running buffer for 10 min. For fresh capillary conditioning, the same rinsing sequence was used but the duration of each step was doubled. In other cases, deionized water, the running buffer, the amine regenerator solution (amine capillary), and the initiator solution (cationic layer, dynamically coated silica capillary) were used for rinsing, with application of the rinsing times as recommended by the manufactures.

Two separation buffers were tested, a borate buffer (Na_2_B_4_O_7_ · 10H_2_O–HCl) of 50 mM ionic strength, pH 9.0, and a phosphate buffer (Na_2_HPO_4_–NaH_2_PO_4_) of 50 mM ionic strength, pH 8.0, each containing 0.4% (v/v) lauryldimethylamine *N*-oxide (LDAO). LDAO is a nondenaturing zwitterionic surfactant with a critical micelle concentration of around 1–2 mM. In dynamic coating with the CEofix^TM^ kit, borate (pH 9.0) and phosphate (pH 8.0) buffers containing an accelerator (polyanion) and 0.4% LDAO were used. Their exact ionic strength could not be determined precisely, but during the runs they both gave currents similar to the ones seen in the buffers of 50 mM ionic strength. The isolation of chlorophyll a (Chla) and Chlase was done as previously [27, 28]; see the electronic supplementary material for more details.

The analysis of the electropherograms, including the peak deconvolution and fitting with a Gaussian function, was done with Origin 9.1 (OriginLab, Northampton, MA, USA). The peak analyzer function with manual baseline definition was used.

### Enzymatic assay

The hydrophobic character of the reaction and separation environment, which is required in the assay of a water-insoluble membrane enzyme interacting with a hydrophobic substrate, was maintained by the use of a zwitterionic surfactant—LDAO. At basic pH, around 9.0, the micellar phase of LDAO provides an optimal medium for both the solubilization of reactants and separation between the substrate (Chla) and the product (chlorophyllide *a*, Chlidea) after application of a voltage [23]. Hence, this separation method is an example of micellar electrokinetic chromatography (MEKC).

Reactions in the off-line format were performed in PCR-type microtubes placed directly on a sample tray, and conditioned inside the CE instrument at 30 °C. The reaction mixture (total volume of 50 μL) was prepared by addition of a 10-μL aliquot of Chla in acetone and a 5-μL aliquot of purified Chlase in LDAO solution to the borate buffer (Na_2_B_4_O_7_ · 10H_2_O–HCl) of 20 mM ionic strength, pH 9.0, containing 1.0% (v/v) LDAO. We recently showed that in these conditions Chlase maintains its catalytic activity [23]. The final concentration of the substrate was 100 μM and that of the enzyme was 2.0 μg/mL The electrophoretic analysis was performed by several consecutive injections of the reaction mixture into the capillary, with application of a forward pressure of 3.45 kPa (0.5 psi) for 5 s, and separation of Chla and Chlidea by subsequent application of a 30-kV voltage. Normal polarity (anode at the inlet) was applied in all cases, except with the amine capillary, which generated the reverse EOF. All reactions with the same capillary were performed in triplicate, with least nine separations being performed in total.

**Fig. 2:**
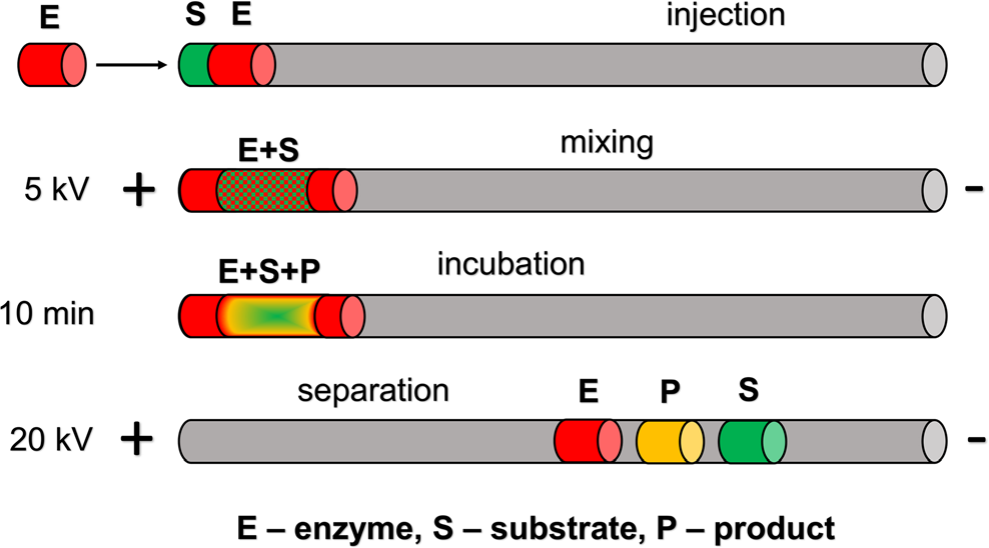
The electrophoretically mediated microanalysis approach, including the four sequential steps (see the text for details)

Reactions in the on-line format were performed with an EMMA in a sandwich-like plug–plug mode [24, 25], achieved by consecutive injection (3.45 kPa/0.5 psi) of three plugs: enzyme (10 s), substrate (5 s), and enzyme (10 s). The concentrations of reactants in the plugs were the same as in the reaction mixture used in the off-line approach. After injection, a 5-kV voltage was applied for 1 min to mix the reactant plugs, and then the zero potential amplification step was applied for 10 min, during which the capillary content was incubated (no voltage). Then a 20-kV separation voltage was applied and the mixture was separated as in the off-line mode. A schematic illustration of all the aforementioned steps is shown in Fig. 2.

### Evaluation criteria

The following criteria were used in the evaluation of the capillaries: reaction progress, selectivity, resolution, theoretical number of plates for Chla (*N*_Chla_), theoretical number of plates for Chlidea (*N*_Chlidea_), and electroosmotic mobility (μ_EOF_).

1 $$ RP=\frac{A_{Chlidea}}{A_{Chlidea}+{A}_{Chla}}, $$

where RP is the reaction progress, *A*_Chlidea_ is the peak area of the product (Chlidea), and *A*_Chla_ is the peak area of the substrate (Chla). Equal values of the molecular absorption coefficients for Chla and Chlidea were used, as previously [23].

2 $$ Sel=\frac{t_{slower}}{t_{faster}}, $$

where Sel is the selectivity and *t*_slower_ and *t*_faster_ are the migration times (min) of the slower and the faster analyte, i.e., Chla or Chlidea respectively, depending on the capillary type.

3 $$ Res = \frac{2\left(\left|{t}_{Chla}-{t}_{Chlidea}\right|\right)}{w_{Chla}+{w}_{Chlidea}}, $$

where Res is the resolution, *t*_*Chla*_ and *t*_Chlidea_ are the migration times (min) of Chla and Chlidea respectively, and *w*_Chla_ and *w*_Chlidea_ are the respective peak widths (min).

4 $$ {N}_{Chla}=5.54{\left(\frac{t_{Chla}}{{w_{1/2}}_{Chla}}\right)}^2, $$

5 $$ {N}_{Chlidea}=5.54{\left(\frac{t_{Chlidea}}{{w_{1/2}}_{Chlidea}}\right)}^2, $$

where *w*_*1*/*2Chla*_ and *w*_1/2Chlidea_ are the respective peak widths at half the peak height (min).

6 $$ {\mu}_{EOF}=\frac{L_{eff\cdot }{L}_{tot}}{U\cdot {t}_{EOF}}, $$

where *L*_eff_ and *L*_tot_ are the effective and total capillary lengths (m) respectively, *U* is the separation voltage (kV), and *t*_EOF_ is the migration time of acetone (min).

## Results and discussion

### Preliminary capillary selection

**Fig. 3:**
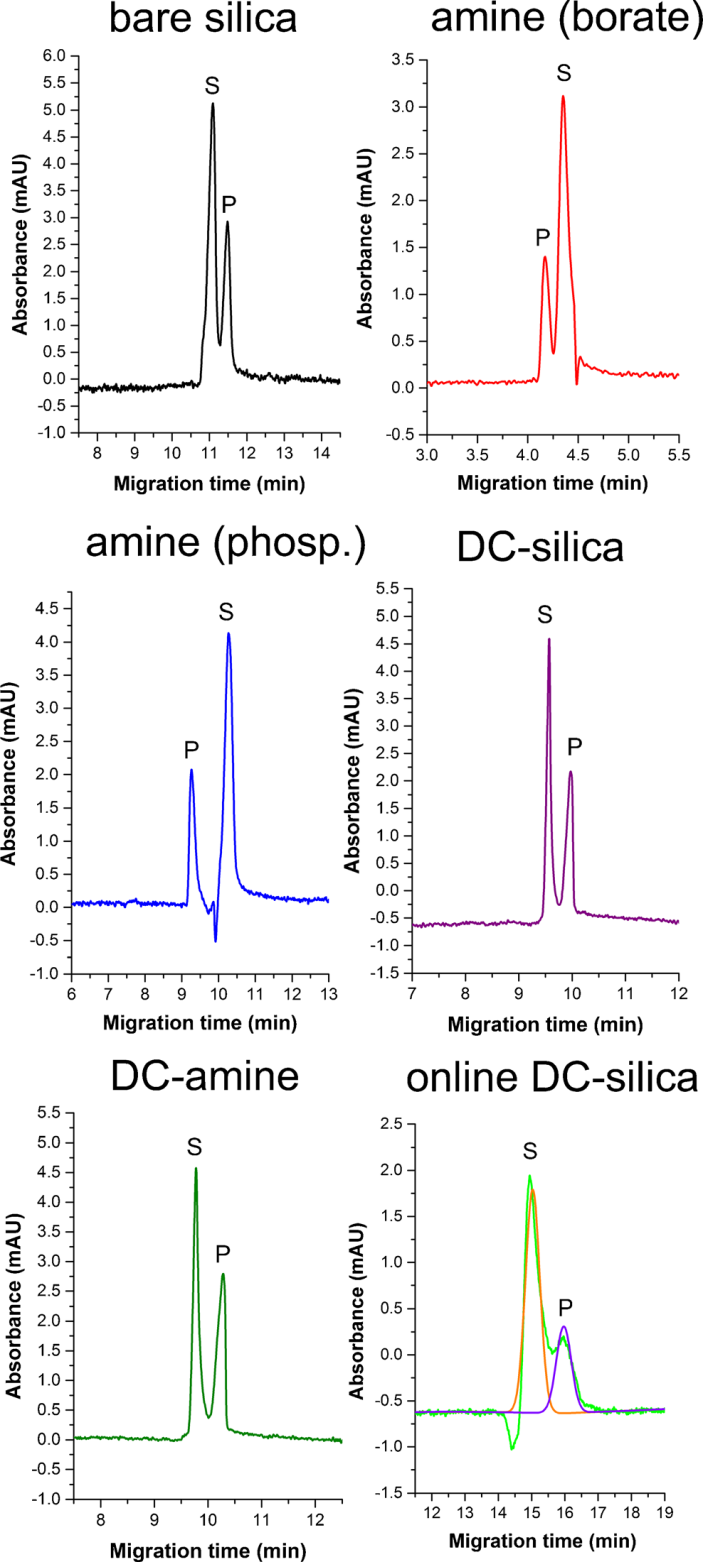
Electropherograms presenting separations between chlorophyll *a* (substrate, *S*) and chlorophyllide *a* (product, *P*) in various capillaries at 430 nm, and the peak deconvolution performed for the on-line reaction. *DC* dynamically coated, *phosp*. phosphate

At the beginning a preliminary test was performed to select the capillaries that gave acceptable resolution between the substrate and the product. The separation enabling reaction monitoring in the conventional off-line reaction mode was achieved with the bare silica capillary, the amine capillary, and the same capillaries subjected to dynamic coating by a polyanionic layer. In most cases the borate buffer was the more effective separation medium, except for the amine capillary, for which the phosphate buffer also gave entirely satisfactory separation. For further tests we selected five separation systems: three capillary types used with borate buffer (uncoated and two dynamically coated), and one type (amine) used with both effective buffers. The respective electropherograms are shown in Fig. 3, and the negative results obtained for the neutral and stationary-phase-coated capillaries are described and discussed in the electronic supplementary material.

The differences between the migration times obtained for Chla and Chlidea, shown in Fig. 3, suggest a similar mechanism of separation in all capillaries. The separation is most likely possible because of selective interactions with the micellar phase, and because of differences in the net charges on the analyte molecules. Chla, being insoluble in water, is expected to have stronger affinity for micelles than water-soluble Chlidea, a carboxylic acid ionized at neutral/basic pH. This weaker binding to micelles entailing a smaller hydrodynamic radius and weaker resistance during migration, and the negative charge, collectively result in higher negative electrophoretic mobility in the normal polarity. Thus, the Chlidea migration time is longer than that of Chla. In the amine capillary, the reverse polarity is applied, and the migration order is reversed.

### Off-line mode

**Fig. 4:**
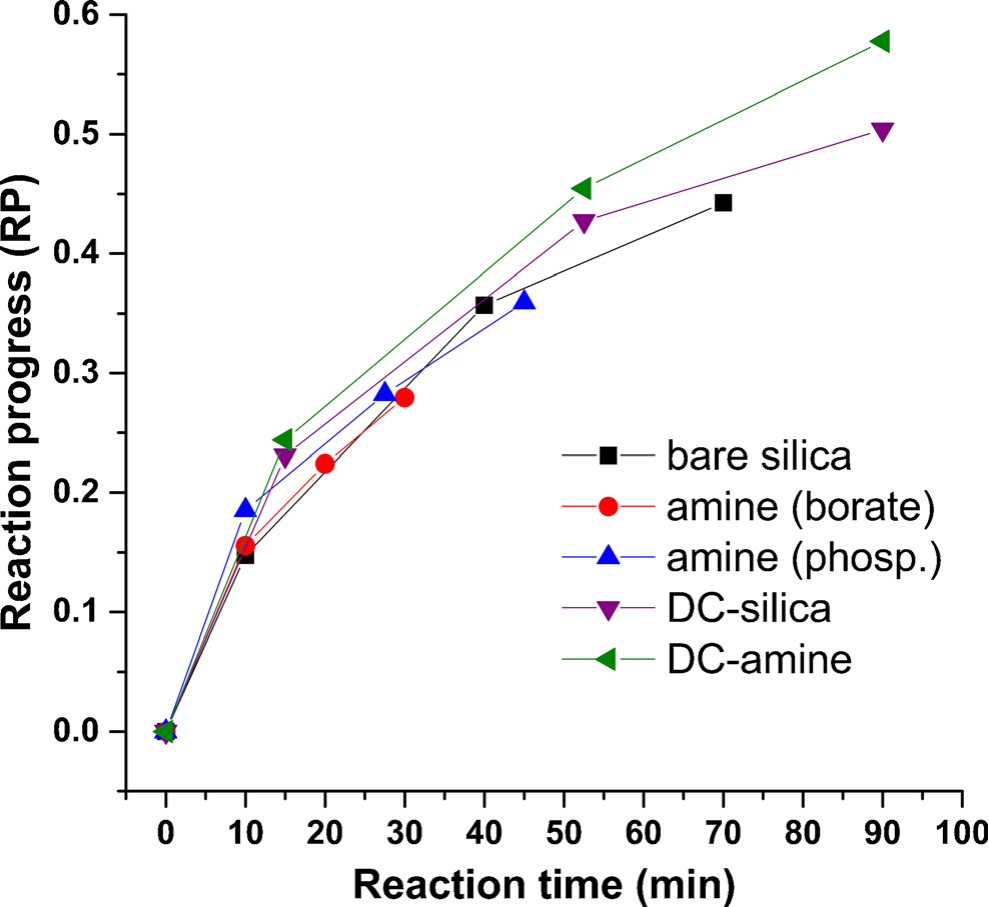
The average reaction progress analyzed off-line in various capillaries (see the text for details). *DC* dynamically coated, *phosp*. phosphate

To estimate the activity of enzyme in various capillaries with the off-line approach, the reaction progress was analyzed at three time points corresponding to three consecutive injections performed from the same vial, placed on the sample tray. The courses of the reactions are depicted in Fig. 4. They are similar, with a characteristic hyperbola-like shape probably reflecting depletion of the substrate and/or an inhibitory effect of the reaction products on the enzyme. It cannot be the effect of acetone because the enzyme is known to be active at much higher concentrations of organic solvents [18, 19]. The close similarity of the plots obtained for the bare silica, the permanently coated, and the dynamically coated capillaries can be taken as an indication of their usefulness in the CE-based off-line assay of Chlase.

The separation between Chla and Chlidea was assessed in terms of the overall precision, defined as the repeatability of the migration times and peak areas. The respective coefficients of variation were determined separately for Chla and Chlidea in each of the evaluated separation systems (Table 1).

**Table 1:** General comparison of all capillaries useful in chlorophyllase off-line and on-line assays on account of the various analytical and practical criteria

Parameter	Bare silica	Amine (borate)	Amine (phosphate)	DC silica	DC amine	On-line DC silica	On-line DC amine
CV_*t*-Chla_	5.8%	2.4%	1.5%	4.9%	1.9%	5.0%	2.9%
CV_*t*-Chlidea_	5.8%	2.4%	1.4%	4.7%	2.0%	5.1%	2.8%
CV_*A*-Chla_	10.2%	5.8%	3.0%	7.6%	7.7%	11.0%	9.8%
CV_*A*-Chlidea_	10.9%	5.7%	3.6%	7.1%	7.8%	12.9%	12.1%
Sel	1.03	1.04	1.09	1.04	1.03	1.04	1.04
Res	1.09	1.13	2.02	1.35	1.72	1.33^a^	1.55^a^
*N*_Chla_	17,202	11,186	7335	25,368	28,786	1092^a^	1140^a^
*N*_Chlidea_	17,802	15,345	9543	26,789	30,120	1157^a^	1231^a^
*H*/IN	1.00 (ref)	0.48	0.73	1.21	1.26	0.57	0.58
μ_EOF_ (10^-4^ m^2^ kV^-1^ min^-1^)	6.8	22.7	8.9	9.2	10.1	9.3	10.0
pH range	Wide	Medium	Medium	Narrow	Narrow	Narrow	Narrow
Conditioning	Medium	Medium	Medium	Long	Medium	Long	Medium
Cost	Low	Medium	Medium	Medium	High	Medium	High

The values presented (except for the coefficients of variation) are the average from all separations.

*CV*_*t*-*Chla*_ and *CV*_*t*-*Chlidea*_ coefficients of variation for the migration times of the substrate (chlorophyll *a*, *Chla*) and the product (chlorophyllide *a*, *Childea*) respectively (*n* = 9); *CV*_*A*-*Chla*_ and *CV*_*A*-*Chlidea*_ coefficients of variation for the peak areas of Chla and Chlidea respectively (*n* = 9); *Sel* selectivity-related parameter, ratio of the migration times obtained for Chla and Chlidea (slower one to faster one); *Res* resolution; *N*_*Chla*_ number of theoretical plates obtained for Chla; *N*_*Chlidea*_ number of theoretical plates obtained Chlidea; *H*/*IN* signal-to-noise ratio measured from the peak height (*H*) and instrumental noise (*IN*) level (mAU) with reference to the bare silica capillary (the value obtained for the reference capillary was assumed to be 1.00 and the values obtained for other capillaries were recalculated accordingly); μ_*EOF*_ electroosmotic mobility/strength of electroosmotic flow; *DC* dynamically coated

^a^The values obtained after peak deconvolution

Concerning the repeatability of migration times, the bare silica capillary gives coefficients of variation of around 6%. This is generally a poor outcome compared with other separation techniques regarded as repeatable (e.g., high-performance liquid chromatography). However, a significant improvement is achieved with the amine capillary, including both the permanently coated inner surface (the borate and phosphate buffers) and the surface dynamically coated by a polyanionic layer(dynamically coated amine). Noticeably, the coefficient of variation in these cases is nearly two to three times lower than for the uncoated silica capillary. It is interesting that the dynamically coated silica capillary gives more than two times higher coefficients of variation than the dynamically coated amine capillary.

The coefficients of variation determined for peak areas are around two times higher than those for the migration times; for the bare silica capillary they are 10–11%. However, again, the permanently coated amine capillary brings a significant improvement, as the coefficients of variation drop to 3–6%. In the case of dynamically coated capillaries, only a weak improvement is noted (coefficient of variation 7–8%). On that account, the use of the permanently coated amine capillary seems to be the best way of increasing the overall precision of the assay. For the amine capillary the repeatability of both the migration times and the peak areas is noticeably better in the phosphate buffer (the lowest coefficients of variation) than in the borate buffer.

The selectivity expressed as a ratio of the migration times of Chla and Chlidea is comparable in each case when the borate buffer is applied (1.03–1.04), and it increases to 1.09 for the amine capillary used with the phosphate buffer. This result and the fact that the phosphate buffer was effective only in the case of the amine capillary point to some coating-specific alteration of the separation mechanism. According to the information provided by the manufacturer (Beckman Coulter), the components of the phosphate buffer may interact selectively with the inner capillary surface by pairing the positive charge of the amine groups [29], causing a partial reduction of EOF and a better separation efficiency than with other buffers. This notion is supported by the assessment of selectivity and resolution (Table 1), as well as by the estimation of the electroosmotic mobility. The electroosmotic mobility calculated on the basis of the acetone peak is more than twofold higher for the amine capillary used with the borate buffer (22.7 cm^2^ · kV^-1^ · min^-1^) than with the phosphate buffer (8.9 cm^2^ · kV^-1^ · min^-1^). This difference in electroosmotic mobility is nicely reflected in the corresponding migration times (Fig. 3). A much weaker EOF may also explain the lower coefficients of variation and better precision seen for the phosphate buffer. This shows that in the case of the amine capillary the use of phosphate buffer may lead to favorable coating-specific alterations of the separation mechanism and to better precision, but results in longer migration times and slower analysis.

The resolution in the permanently coated amine and dynamically coated amine and silica capillaries is better compared with that in the bare silica capillary (Table 1), and reaches a higher value for the dynamically coated amine capillary than for the dynamically coated silica capillary. The theoretical number of plates expresses peak efficiency, and the highest values were obtained for the two dynamically coated capillaries, and they are 50% higher than for the uncoated silica capillary. The narrower and well-resolved peaks may facilitate prospective extension/enrichment of the method with additional reaction components, giving a better separation of the analytes. At the same time, the performance of the permanently coated amine capillary is worse in both cases than that of the bare silica capillary.

The average total peak height of Chla and Chlidea obtained for the uncoated silica capillary is greater than for the other capillaries, but the permanently and dynamically coated capillaries, in turn, generate significantly lower instrumental noise (not shown). In principle, the signal-to-noise ratio is perhaps a more appropriate criterion for capillary evaluation as it is related to the limits of detection and quantification, which are important in the assays of enzymes of low activity or in inhibition studies. The permanent coating by the amine layer significantly reduces the signal-to-noise ratio, by 50%, whereas the dynamic coating increases it by around 20% (Table 1). Therefore, for the detection and quantification of low amounts of reaction products, dynamically coated silica or amine capillaries seem the best choice.

As mentioned earlier, in capillary evaluation the strength of EOF in another useful parameter that determines migration times and the speed of analysis. Except for the amine capillary, the strength of EOF was very similar. The increase in EOF seen on dynamic coating of the silica capillary was weak. However, EOF was more than twice as strong in the amine capillary used with the borate buffer as in the bare silica capillary, similar to the results obtained by us with buffers devoid of a micellar phase [26]. This suggests that the amine capillary is generally a good choice for both the significant reduction of migration times and the improvement of their repeatability.

Additional criteria that may be useful in a general characterization and evaluation of various capillaries are (1) the range of pH that can be applied without detriment to the coating (recommended by the manufacturer), (2) the simplicity of the rinsing/conditioning procedure, including a recommended number of flushing solutions required to achieve suitable reproducibility of the inner surface, and (3) the cost of a particular capillary modification (Table 1). The use of dynamically coated capillaries is restricted to buffers of relatively narrow pH range. The reagents for dynamic coating are offered as ready to use, without the possibility of preparing them de novo, and the modification of pH is very limited. Secondly, the dynamic coating applied to the amine capillary is simpler than that applied to the bare silica capillary because the step involving flushing with the polycation is unnecessary (the amine capillary is itself positively ionized). In terms of economy, this variant though is the most expensive because the cost of the amine capillary adds to the cost of the dynamic coating kit.

### Electrophoretically mediated microanalysis

In the EMMA approach the bare silica and permanently coated amine capillaries were ineffective: the peaks of the analytes strongly overlapped, which hampers the analysis (not shown). The most probable reason is that the prolonged time spent by the analytes inside the capillary causes larger diffusional effects. The analysis of Chlase activity in this mode was feasible only with the dynamically coated silica and amine capillaries, but because of a drop in resolution (Fig. 3), a peak deconvolution was required to estimate the RP values. The average value RP was then 0.28–0.29 for both the dynamically coated silica capillary and the dynamically coated amine capillary. Surprisingly, assuming that the effective concentrations of substrate and enzyme were the same in both modes, this is more than 180% of the reference values obtained after 10 min in the off-line mode in the same capillaries. In the EMMA approach, however, it is difficult to precisely determine the in-capillary total reaction time. Although the incubation lasts 10 min, the substrate–enzyme contact time is probably somewhat longer. The enzyme plug may remain partially mixed with the substrate plug during separation over some period, because of the similar migration velocities. According to the known amino acid sequence of Chlase [30, 31], one may expect the lack of net charge of Chlase and Chla at neutral/weakly basic pH and similarly the hydrophobic character of both molecules to cause the strong affinity for the micellar phase. Therefore, despite the mobility of the enzyme being unknown (no signal was detected), it is most likely comparable to the that of the substrate. Thus, it seems reasonable to assume that the *RP* values obtained confirm a reaction time longer than the nominal 10 min. On the other hand, the total amount of enzyme injected into the capillary with respect to the substrate was larger than in the off-line mode, as follows from the length of the injection zones. Therefore, after diffusional mixing, the enzyme-to-substrate ratio could be increased in comparison with that in the off-line reaction. Hence, although activity of the enzyme can be analyzed by EMMA with the zero potential amplification step, further optimization and calibration will be necessary to apply both reaction modes interchangeably. The introduction of an in-capillary incubation adversely affects the resolution between the analytes; however, the dynamic coating providing better peak efficiency appears to be an effective means for counteracting this undesirable effect and for maintaining sufficient resolution for peak deconvolution.

The repeatability of migration times is generally similar in both reaction modes, whereas the coefficients of variation obtained for the peak areas are noticeably larger in EMMA than in the off-line mode (Table 1). This could be due to some unfavorable effects inherent to in-capillary incubation (e.g., an increased dispersion of the injection zones). An additional source of random errors could be the peak deconvolution. In the on-line mode the signals from Chla and Chlidea largely overlapped in all cases, except for the dynamic coating, where the peaks were better resolved. The resolution for the deconvoluted peaks is quantitatively very similar as in the same capillaries used in the off-line mode.

However, the analysis based on deconvolution results in a significantly reduced number of theoretical plates, even more than 20 times in comparison with the same capillaries used in the off-line reaction mode (Table 1). This stems most likely from the aforementioned stronger diffusional effects, causing peak broadening due to the incubation performed in-capillary. This also explains the lower signal-to-noise ratio.

## Conclusions

In this work we have shown the applicability of both permanent and dynamic capillary coatings in optimization of the MEKC off-line and on-line assays of an experimentally demanding membrane enzyme. The comparison of a variety of criteria shows that the permanently coated amine capillary (eCAP) and the dynamically coated silica and amine capillaries (CEofix), used instead of the conventional uncoated silica capillary, give significant analytical improvements. The use of a permanently coated amine capillary, particularly in the case of phosphate buffer, appreciably enhances the repeatability of the migration times and peak areas, and this makes CE competitive with other analytical techniques. This type of coating may also favorably change the separation efficiency obtained with phosphate buffer, or speed up analysis with borate buffer. The dynamic coating, most importantly, facilitates a switch between the off-line and on-line assay modes. Despite the zero potential amplification step in the on-line mode, the resolution of analytes remains sufficient to assess reaction progress via peak deconvolution. In the off-line mode, the peak efficiency and signal-to-noise ratio are also enhanced by dynamic coating. It is worth emphasizing that the permanently coated amine capillary may be used as a matrix for dynamic coating, and the outcomes seen in this case appear even better than for the dynamically coated bare silica capillary. This specific double coating ensures the increased repeatability of migration times and requires one step less during the capillary conditioning. Its disadvantage lies in its high cost, as it requires the purchase of both an amine capillary and a dynamic coating kit. In addition, in principle the dynamic coating may be applied to capillaries of any dimensions, whereas the permanently coated capillaries are available in only one or two variants of internal diameter. These features make both permanently and dynamically coated capillaries a potentially attractive choice for enzymatic assays using CE, separations in micellar media, and other applications of the CE technique.

## Electronic supplementary material

Below is the link to the electronic supplementary material.

ESM 1 (PDF 265 kb)

## Abbreviations

CE: Capillary electrophoresis
Chla: Chlorophyll *a*
Chlidea: Chlorophyllide *a*
Chlase: Chlorophyllase
EMMA: Electrophoretically mediated microanalysis
EOF: Electroosmotic flow
LDAO: Lauryldimethylamine *N*-oxide
MEKC: Micellar electrokinetic chromatography
