# Supplementary material for: Capillary coating as an important factor in optimization of the off-line and on-line MEKC assays of the highly hydrophobic enzyme chlorophyllase
Source: Anal Bioanal Chem. 2016 Nov 25;409(6):1493–501. doi: 10.1007/s00216-016-0097-5 (PMC5306352; doi:10.1007/s00216-016-0097-5)
Supplement: Supplementary file 1 — (PDF 265 kb) [file 216_2016_97_MOESM1_ESM.pdf]

Analytical and Bioanalytical Chemistry

Electronic Supplementary Material

Capillary coating as an important factor in optimization of the off-line and on-line MEKC assays of a highly hydrophobic enzyme chlorophyllase

Paweł Mateusz Nowak, Michał Woźniakiewicz, Maciej Michalik, Leszek Fiedor,
Paweł Kościelniak

Preparation of chlorophyll

Chlorophyll a (Chla) was isolated from frozen spinach and separated from chlorophyll b by column chromatography on a DEAE-Sepharose (Pharmacia) equilibrated with n-hexane and 1.5% (v/v) of 2-propanol in n-hexane as the eluent. The final purification was done via isocratic RP-HPLC on a semi-preparative C-18 column (Microsorb 100-5, 250×10 mm diameter, Varian), using methanol as the eluent at the flow rate of 4 ml/min. The separation was performed with a Pro Star 240 pump system (Varian) coupled to a DAD detector (TIDAS, J&M) for the online detection of absorption spectra. Only the S diastereoisomer (non-prime epimer) of Chla with a retention time of 24.2 min was collected. All preparative steps were carried out under dim light to minimize pigment degradation.

Preparation of chlorophyllase

Cloning of CLH1 gene

The total RNA was isolated using Chomczynski method [1] from leaves of 6 weeks old *Arabidopsis thaliana* WT Columbia plants illuminated for 3 hours with white light. The cDNA was synthesized with the First Strand cDNA Synthesis Kit (Fermentas) using random hexamer primers. The full coding sequence of CLH1 was amplified by polymerase chain reaction (PCR) using an Easy-A High Fidelity PCR cloning Enzyme (Stratagene) with the primers containing extra underlined restriction sites:

5'-GAATTCGCGGCGATAGAGGACAGTCCAACG-3'; (CLH1 forward EcoRI)

5'-CCCGGGGACGAAGATACCAGAAGCTTCTTCCAACTCAGGTG-3'; (CLH1 reverse XhoI)

The PCR products were cloned into the pGEM®-T Easy Vector (Promega) and sequenced. The fragments obtained after digestion with EcoRI/XhoI for pGEM-T-Easy-CLH1 were cloned into the pGEX4T1 expression vector which contained the GST-tag (Amersham Biosciences). The constructs: pGEX4T1-CLH1 and empty pGEX4T1 (control) were then transformed into the E. coli strain BL21.

Expression and isolation of CLH1 proteins

The CLH1 expressing E. coli strains were cultivated using liquid Luria-Bertani (LB) medium and ampicillin (100 µg/ml) as a selecting factor. Bacterial culture was started from a single colony, incubated overnight at 37°C in 75 ml LB medium, and passaged to 750 ml of LB medium. After 2 h of incubation, isopropyl β-D-1-thiogalactopyranoside (IPTG) at 1 mM was added to induce the

expression of CLH1 fusion protein, and after another 2 h of incubation at 27°C each batch was centrifuged for 15 min at 4500 g and pooled.

Bacterial pellet was suspended in 5 ml of PBS buffer (20 mM phosphate buffer, 150 mM NaCl, pH 7.6) and homogenized with a Potter-type teflon grinder. Then, dithiothreitol (DTT) at 2 mM was added, and bacteria cells were disrupted using a Cole Palmer 4710 ultrasonic homogenizer for 5 minutes at 4°C. After addition of lauryldimethylamine-oxide (LDAO) to 0.2%, the mixture was stirred for 2 h at ambient temperature, to release CLH1 protein from inclusion bodies. Insoluble cell debris was removed by centrifugation for 15 min at 20000 g, and the supernatant was subjected to affinity chromatography on a glutathione agarose (Sigma-Aldrich) column coupled to a low pressure gradient pump. The separation was run at 4°C with 1 ml/min flow, and protein elution was monitored at 280 nm with UV detector. The CLH1 fusion protein was bound on a column and rinsed with PBS buffer containing 0.2% LDAO, until a complete elution of contaminating proteins. Then, the fusion protein was digested overnight with 1 U of human plasma thrombin (Sigma-Aldrich), and afterwards the column was again rinsed with PBS buffer and 0.2% LDAO. The quality of fraction containing eluted protein was assessed by SDS-PAGE on a 12% resolving gel in a buffer system of Laemmli [2]. Finally, CLH protein was quantified using BCA assay kit (Novagen) and stored at -30°C until use.

Negative results

No analytes were detected in the neutral capillary, even after applying a strong forward pressure to speed up the separation (not shown). A significantly lower peak detected for acetone (EOF marker) at 220 nm suggests that the lack of absorption of light at 430 nm (the Soret bands of analytes) is caused by a general drop of sensitivity in this capillary rather than by an irreversible adsorption of analytes on the capillary inner surface. It is likely that this particularly low sensitivity arises from a strong absorption of light by the polyacrylamide coating, especially in the region near the analytical wavelength (430 nm). The signals from Chla and Chlidea were also not found in the C18 capillary column, but in this case, the hydrophobic interactions between reagents, micelles, and hydrocarbon chains of capillary coating are likely responsible for a strong retention of analytes on the stationary phase and a consequent lack of the signal. This conclusion is confirmed by a relatively strong signal from acetone, as in the bare silica capillary. By contrast, one asymmetric peak was recorded at 430 nm in the diol and cholesterol capillary columns, due to unresolved Chla and Chlidea. This indicates that the diol and cholesterol phases adversely affects the separation mechanism, making these coatings not applicable to Chlase assay.

References

[1] Chomczynski P, Sacchi N. Anal Biochem Academic Press. 1987;162:156–159.

[2] Laemmli U. Nature. 1970;227:680–685.
